# From Pig Breeding Environment to Subsequently Produced Pork: Comparative Analysis of Antibiotic Resistance Genes and Bacterial Community Composition

**DOI:** 10.3389/fmicb.2019.00043

**Published:** 2019-01-29

**Authors:** Zongbao Liu, Uli Klümper, Lei Shi, Lei Ye, Meng Li

**Affiliations:** ^1^Institute for Advanced Study, Shenzhen University, Shenzhen, China; ^2^Key Laboratory of Optoelectronic Devices and Systems of Ministry of Education and Guangdong Province, College of Optoelectronic Engineering, Shenzhen University, Shenzhen, China; ^3^ESI and CEC, Biosciences, University of Exeter, Cornwall, United Kingdom; ^4^European Centre for Environment and Human Health, University of Exeter, Truro, United Kingdom; ^5^Institute of Food Safety and Nutrition, Jinan University, Guangzhou, China

**Keywords:** pig farm, antibiotic resistance genes, bacterial community composition, breeding environment, pork

## Abstract

It is well verified that pig farms are an important reservoir and supplier of antibiotic resistance genes (ARGs). However, little is known about the transmission of ARGs between the breeding environment and subsequently produced pork. This study was conducted to investigate if ARGs and associated host bacteria spread from the breeding environment onto the meat through the food production chain. We thus analyzed the occurrence and abundance of ARGs, as well as comparing both ARG and bacterial community compositions in farm soil, pig feces and pork samples from a large-scale pig farm located in Xiamen, People’s Republic of China. Among the 26 target ARGs, genes conferring resistance to sulfonamide, trimethoprim, aminoglycoside, chloramphenicol, macrolide, florfenicol, and tetracycline were observed at high frequency in both the pig breeding environment and pork. The prevalence of ARGs in pork was surprisingly consistent with breeding environments, especially between the pork and feces. The relative abundance of 10 representative ARGs conferring resistance to six classes of antibiotics ranged from 3.01 × 10^-1^ to 1.55 × 10^-6^ copies/16S rRNA copies. The ARGs conferring resistance to sulfanilamide (*sulI* and *sulII*), aminoglycoside (*aadA*), and tetracycline [*tet(A)* and *tet(M)*] were most highly abundant across most samples. Samples from feces and meat possessed a higher similarity in ARG compositions than samples from the farms soil. *Enterobacteriaceae* found on the meat samples were further identical with previously isolated multidrug-resistant bacteria from the same pig farm. Our results strongly indicate that ARGs can be potentially spreading from pig breeding environment to meat via the pork industry chain, such as feed supply, pig feeding and pork production.

## Introduction

The increasing prevalence and spread of antibiotic resistance genes (ARGs) from food animal sources has become a major public health concern (O’Neill, 2015). Livestock farm environments, such as farmed soils and animal waste, have been considered the most important reservoirs for environmental ARGs, as high abundances of various ARGs have frequently been detected in these environments (Cheng et al., 2013; Zhu et al., 2013; Li et al., 2015; He et al., 2016; Fang et al., 2018; Qian et al., 2018). It is generally accepted that the use of antibiotics in animal husbandry is one of the major drivers for the emergence of resistant bacteria and dissemination of resistance genes. The long-term and extensive use of antibiotics in food animals is not only a regional or national phenomenon, but part of a global problem. In 2010, global consumption of antimicrobials in food animal production was estimated at 63,151 (± 1,560) tons (Van Boeckel et al., 2015). In the United States, livestock producers used between 70% and 80% of all antibiotics sold across the country (Elliott et al., 2017). In Vietnam, more than 11 antibiotics were used for growth promotion, 25 for disease prevention, and 37 for therapeutic purposes in pig farming (Tao et al., 2014). As the largest producer and consumer of antibiotics in the world, China produced approximately 210,000 tons of antibiotics each year, 46.1% were used in the livestock industries (Liu et al., 2015). More than 85% of these administered antibiotics or their metabolites may be excreted through animal urine or feces and then discharged into the environment (Tao et al., 2014). Antibiotics will impose a widespread selective pressure on bacteria, leading to the enrichment of resistant strains, which are also capable of spreading between different environments (Andersson and Hughes, 2014). Furthermore, many ARGs are encoded on mobile genetic elements allowing their transmission upon entering a new environment independent of the original host to a multitude of bacteria from the indigenous community (Klümper et al., 2015). Consequently, bacteria with various ARGs are commonly found in food animal wastes and the ambient environment nearby livestock farms (Tao et al., 2014; Jia et al., 2017). A potential transmission route of these antibiotic-resistant bacteria and ARGs from food animal sources to humans is the meat industry chain.

Currently, main global monitoring efforts focusing on antibiotic consumption and antibiotic-resistant bacteria takes place in clinical and public health laboratories, while they are rarely focused on animal husbandry in most countries, especially in China. However, previous studies have revealed that an exchange of ARGs could occur between bacteria from farm animals/soils and clinical pathogens via horizontal gene transfer (Forsberg et al., 2012; Li et al., 2015). Thus, environments carrying drug-resistant bacteria are indeed potential reservoirs of clinical resistance genes. Therefore, investigating the prevalence, abundance and transmission of antibiotic-resistant bacteria and ARGs on livestock farms is essential for controlling antibiotic resistance. Many studies have examined the abundance of ARGs in pig farm environments using real-time polymerase chain reaction (real-time PCR) (Cheng et al., 2013; Zhu et al., 2013; Tao et al., 2014). However, few studies have determined the relative abundances of ARGs of bacteria residing in or on pork. As far as we know, no study has performed a comparative analysis of the abundances and similarities of ARGs in pig farm soils, pig feces and the subsequently produced meat products. Since the ARG composition is significantly correlated with microbial phylogenetic and taxonomic structure (Forsberg et al., 2014), we here combined the analysis of ARG composition and bacterial community composition to provide a better understanding of the dynamics of ARG transfer between environmental and meat samples.

The objectives of this study were (1) to determine the occurrence and abundance of ARGs in pig farm soil, fecal and meat samples collected from a large-scale pig farm based on PCR and real-time PCR methods; (2) to evaluate the similarity/difference of ARG compositions among different types of samples using non-metric multidimensional scaling (NMDS) analysis; and (3) to analyze the composition of the dominant bacterial community using PCR-denaturing gradient gel electrophoresis (DGGE) analysis.

## Materials and Methods

### Sample Collection

A total of 68 farm soil, pig feces and fresh pork meat were collected from a large-scale pig farm over a period of more than one year (August 2012, April 2013, and November 2013) in Xiamen, China (longitude, 117°59′E; latitude, 24°51′N). On this farm sulfonamides/trimethoprim (trimethoprim is a potentiator that is often administered together with sulfonamides), tetracycline, gentamicin, streptomycin, chloramphenicol, florfenicol, and amoxicillin are used widely for the treatment of swine infections or as growth promoters. However, exact doses of each of the antibiotics were not available from the farm. Twenty-seven surface soil samples (0–8 cm) were collected nearby 27 independent houses of finishing pigs. For each soil sample, three replicates (each 100 g) were collected around one finishing pig house, homogenized and combined into one sample for DNA extraction. Nineteen pig fecal samples were collected from a total of six waste treatment pools approximately 30 m from the pig breeding area using sterile centrifuge tubes. Twenty-two meat samples (approximately 200 g) from different finishing pigs were collected in the slaughter room using aseptic methods and stored at 4°C for a subsequent DNA extraction. All the samples were placed immediately on ice and transported to the laboratory for homogenization and DNA extraction.

### DNA Extraction

The bacterial genomic DNA of the meat samples was extracted according to the following procedures. First, the meat samples (∼200 g) were rinsed with 50 mL of sterile peptone water, and then ∼50 g of each sample was placed aseptically into a sterile lateral filter bag containing 100 mL of 0.1% sterile peptone water, and the following procedures were performed as described previously (Wang et al., 2006). Fifty milliliters of filtered meat homogenate was centrifuged at 500 × *g* for 10 min, and then 20 mL of the supernatant was transferred to another sterile centrifuge tube and centrifuged at 14,000 × *g* for 10 min; the precipitate was used for DNA extraction using the Mag-MK Bacterial Genomic DNA Extraction Kit (Sangon, China). The genomic DNA of the soil and fecal samples was extracted using the PowerSoil DNA Isolation Kit (Mo Bio, Germantown, MD, United States) according to the manufacturer’s instructions. The quality and concentration of the DNA were determined by spectrophotometer analysis (NanoDrop ND-1000C, Thermo Fisher Scientific, United States), low-purity DNA (with *A_26_*_0_/*A_280_* ratio < 1.6 or > 2.0, or *A_260_*/*A_230_* ratio < 1.8) was further purified using Dr. GenTLE Precipitation Carrier Kit (Takara, Shiga, Japan).

### PCR Detection of ARGs

Twenty-six ARGs were analyzed using a PCR assay; the primers used are listed in Supplementary Table S1. The PCRs were performed in a total volume of 25 μL including 1 μL of extracted DNA, 2.5 μL of *Taq* reaction buffer, 0.2 mM dNTPs, 0.2 μM primers, and 0.625 units of Hot Start *Taq* DNA polymerase (Takara, Shiga, Japan). The PCR conditions were as follows: 95°C for 3 min, followed by 30 cycles of 94°C for 0.5 min, 55–60°C for 0.5 min, and 72°C for 1 min, followed by one cycle of 72°C for 10 min. The PCR products were analyzed with electrophoresis on 1.5% agarose gels in 1 × Tris–acetate–EDTA buffer (40 mM Tris, 20 mM acetic acid, and 1 mM EDTA, pH 8.0) at 100 V for 30 min.

### Real-Time PCR Detection of ARGs and 16S rRNA Genes

The real-time PCR analyses were performed on an ABI 7500 instrument (Applied Biosystems, Foster City, CA, United States) to quantify the copy number of the *sulI, sulII, aadA, aphA-1, cmlA, ermB, floR, tet(A), tet(B), tet(M)* genes, as well as the 16S rRNA V3 region. Standard curves for the real-time PCR assays were generated as described previously (Colomer-Lluch et al., 2011). Recombinant plasmids containing the target genes were used as positive controls. To construct the recombinant plasmids, the target ARGs and 16S rRNA V3 region gene were amplified with PCR and cloned into the pBackZero-T vector (Takara), and verified by sequencing at the Sangon Biological Engineering Technology & Service Company (Shanghai, China). The real-time PCRs were performed in a total volume of 25 μL using the SYBR Premix *Ex Taq* (Tli RNaseH Plus) Kit, including 1 μL of extracted DNA and 0.2 μM of each primer. The real-time PCR conditions were as follows: 95°C for 2 min, followed by 40 cycles of 95°C for 15 s, 57–60°C for 30 s, and 72°C for 45 s, followed by a melting curve stage.

### PCR-DGGE Analysis of Dominant Bacterial Community

The V3 variable region of 16S rRNA genes was used to analyze the composition of the dominant bacterial community. First, the 16S rRNA genes were amplified from the genomic DNA by PCR using primers 27F (5′-AGAGTTTGATCCTGGCTCAG-3′) and 1492R (5′-GGTTACCTTGTTACGACTT-3′) as described previously (Liu et al., 2015). Then, the PCR product was purified using TaKaRa MiniBEST DNA Fragment Purification Kit (Takara) according to the manufacturer’s recommendations and diluted to 50 ng/μL with sterile double-distilled water. The concentration and purity of the DNA was checked with a NanoDrop 2000c spectrophotometer (Thermo Fisher Scientific, Waltham, MA, United States). Subsequently, the V3 variable region for the DGGE analysis was amplified from the purified 16S rRNA genes with PCR using the primers 338F-GC (5′-CCTACGGGAGGCAGCAG-3′) and 518R (5′-ATTACCGCGGCTGCTGG-3′) (Zhang et al., 2016). To increase the stability of DGGE, a GC clamp (CGCCCGCCGCGCGCGGGGGCGGGGCGGGGGCACGGGGGG) was added to the 5′ end of the primer 338F (Muyzer et al., 1993). The PCR was performed in a total reaction volume of 50 μL containing 1 μL of 50 ng/μL purified 16S rRNA genes, 5 μL of *Taq* reaction buffer, 0.2 mM dNTPs, 0.2 μM primers, and 1.25 units of Hot Start *Taq* DNA polymerase. A touchdown PCR was used to amplify the 16S rRNA gene V3-GC region to increase the specificity of the amplification. The program was performed as follows: an initial denaturation at 94°C for 3 min, followed by 10 cycles of 94°C for 30 s, 65°C for 30 s with a 1°C decrease per cycle, and 72°C for 1 min, followed by 25 cycles of 94°C for 30 s, 55°C for 30 s, and 72°C for 1 min, followed by one cycle of 72°C for 10 min. The amplified products were confirmed by gel electrophoresis.

The DGGE analysis of the 16S rRNA V3-GC regions was performed on a DCode System apparatus (Bio-Rad, Hercules, CA, United States) as described by Muyzer and Smalla (Muyzer et al., 1993; Muyzer and Smalla, 1998). PCR samples were separated on 8% acrylamide gels with an optimal denaturing gradient. To optimize the denaturing gradient, DGGE for each type of sample was performed using denaturant gradients of 35–65, 40–60, 45–60, and 40–55%. Based on these preliminary results, the linear gradient of 40–60% denaturant was chosen to analyze the meat samples. For the soil and fecal samples, denaturant gradients of 45–60 and 40–55% were used, respectively. Electrophoresis was performed in 1× Tris–acetate–EDTA buffer at a constant voltage of 60 V and 60°C for 16 h. After electrophoresis, the gels were incubated in ethidium bromide solution for 30 min and rinsed with double-distilled water for 10 min. Images of the gels were obtained using the GelDoc XR System (Bio-Rad) according to the manufacturer’s instructions. For each DGGE lane, band number and position were assessed for pattern similarity using Quantity One image analysis software (Bio-Rad, Hercules, CA, United States).

### Identification of DGGE Bands

The most detected and obvious DGGE bands were marked from each acrylamide gel. The bands were excised carefully from the acrylamide gels using a sterile scalpel. Every excised band was briefly washed tree times with 1 mL of double-distilled water in a 1.5-mL sterile centrifuge tube, and then crushed by a pipette tip. DNA fragments in crushed bands were eluted with 50 μL of double-distilled water by incubating overnight at 4°C. The dissolved solution was centrifuged at 12,000 × *g* for 10 min, and the liquid supernatant was used as the template for reamplification of the PCR products with primer 338F without a GC clamp and primer 518R. The PCR conditions were as follows: 95°C for 3 min, followed by 30 cycles of 94°C for 30 s, 55°C for 30 s, and 72°C for 1 min, followed by one cycle of 72°C for 10 min. The PCR products were cloned into the pBackZero-T vector and sequenced at the Sangon Biological Engineering Technology & Service Company. All DGGE band sequences were shown in the Supplementary File.

### Statistical Analysis

Non-metric multidimensional scaling was used to visualize the similarity of the ARG compositions in the 40 soil, fecal and meat samples. NMDS was performed using the abundance correlation matrix of the ARGs. Furthermore, differential abundance of ARGs between environmental and meat samples was tested by one-way analysis of variance (ANOVA). All statistical analyses were performed with Paleontological STatistics (PAST) software (version 3.16). Sequence identity was analyzed by comparison with GenBank sequences using the Basic Local Alignment Search Tool program^1^. Sequences with 97% or higher identity were considered to represent the same species. MEGA 6.06 (Center for Evolutionary Functional Genomics, Tempe, AZ, United States) was used to construct the neighbor-joining phylogenetic tree. A phylogenetic analysis based on the V3 region of 16S rRNA gene sequences used the maximum composite likelihood method. A bootstrap analysis was performed using 1000 replicates.

## Results

### Distribution of ARGs

The prevalence of 26 resistance genes in 68 meat and environmental samples was determined by a PCR assay. Genes responsible for resistance to sulfonamide (*sulI* and *sulII*), trimethoprim (*dfrA17*), aminoglycoside (*aadA* and *aphA-1*), chloramphenicol (*cmlA*), a macrolide (*ermB*), florfenicol (*floR*), and tetracycline [*tet(A), tet(B)*, and *tet(M)*] were distributed widely, as they were detected in 100, 100, 54.4, 100, 100, 100, 92.6, 100, 94.1, 80.9, and 92.6% of the samples, respectively (Table 1). Among these, *sulI, sulII, aadA, aphA-1, cmlA*, and *floR* were observed in every single sample. In contrast two genes, *dfrA12* (trimethoprim) and *aadB* (aminoglycoside) had low detection rates. The *dfrA12* gene was found only in meat (31.6%) and fecal samples (22.7%). In contrast, the *aadB* gene conferring resistance to an aminoglycoside was found exclusively in 22.2% of soil samples. Four tetracycline resistance genes were chosen as target ARGs in this study, of which, *tet(A)* and *tet(B)* were observed in all meat samples. Contrary, in fecal and soil samples, the resistance gene *tet(M)*, instead of *tet(A)* or *tet(B)*, was detected in every sample (Table 1).

**Table 1:** Statistics of antibiotic resistance genes in all samples.

Antimicrobial		No. (%) of samples			
resistance	Gene	containing resistance genes			
		Soil	Feces	Meat	Total
		(*n* = 27)	(*n* = 19)	(*n* = 22)	(*n* = 68)
Sulfonamide	*sulI*	27 (100)	19 (100)	22 (100)	68 (100)
	*sulII*	27 (100)	19 (100)	22 (100)	68 (100)
Trimethoprim	*dhfrV*	0	0	0	0
	*dhfrI*	0	0	0	0
	*dfrA12*	0	6 (31.6)	5 (22.7)	11 (16.2)
	*df rA17*	7 (25.9)	18 (94.7)	12 (54.5)	37 (54.4)
Aminoglycoside	*aadA*	27 (100)	19 (100)	22 (100)	68 (100)
	*aadB*	6 (22.2)	0	0	6 (8.8)
	*aac(3)-I*	0	0	0	0
	*aphA-1*	27 (100)	19 (100)	22 (100)	68 (100)
	*aac(3)-IV*	0	0	0	0
Chloramphenicol	*catI*	0	0	0	0
	*cmlA*	27 (100)	19 (100)	22 (100)	68 (100)
Beta-lactam	*blaSHV*	0	0	0	0
	*blaOXA*	0	0	0	0
	*blaTEM*	0	0	0	0
AmpC’s	*citM*	0	0	0	0
	*moxM*	0	0	0	0
	*dhaM*	0	0	0	0
Macrolide	*ereA*	0	0	0	0
	*ermB*	23 (85.2)	18 (94.7)	22 (100)	63 (92.6)
Florfenicol	*floR*	27 (100)	19 (100)	22 (100)	68 (100)
Tetracycline	*tet(A)*	24 (88.9)	18 (94.7)	22 (100)	64 (94.1)
	*tet(B)*	14 (51.9)	19 (100)	22 (100)	55 (80.9)
	*tet(M)*	27 (100)	19 (100)	17 (77.3)	63 (92.6)
	*tet(D)*	0	0	0	0

In addition, the detection rate of individual ARGs differed significantly between different batches of samples. For example, among the fecal samples, the *dfrA12* gene conferring resistance to trimethoprim was detected in 100% of the samples in the second batch (collected in April 2013). However, this gene was not observed in the other batches of samples. Similarly, in the meat samples, this gene was only detected in samples of the third batch (collected in November 2013), 71.4% of which tested positive. The tetracycline resistance gene *tet(M)* was detected frequently, as it was found in 100 and 85.7% of the first and third batches of the meat samples, respectively, while it was only detected in 50% of the samples of the second batch (Supplementary Table S2). However, 13 further resistance genes [*dhfrV, dhfrI, aac(3)-I, aac(3)-IV, catI,blaSHV, blaOXA, blaTEM, citM, moxM, dhaM, ereA*, and *tet(D)*] were not detected in any meat or environmental samples.

### Quantification of ARGs and 16S rRNA Gene

**FIGURE 1:**
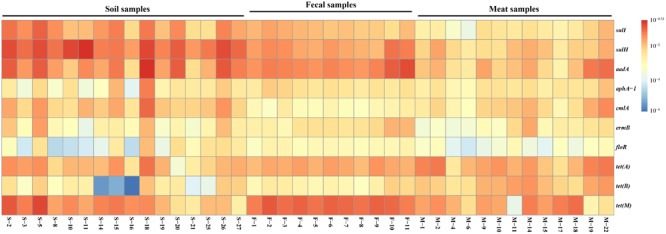
The relative abundance of 10 ARGs in the 40 representative environmental and meat samples.

Based on the previous prevalence testing, 10 representative ARGs were chosen in combination with the 16S rRNA gene and their copy number was determined in 40 representative soil, fecal and meat samples with qualitative real-time PCR assays. Relative ARG abundance (defined as the absolute number of ARG copies normalized to the absolute number of 16S rRNA) was used to compare the differences of 10 ARGs among the different samples. Ten ARGs conferring resistance to six classes of antibiotics were detected with abundances ranging from 1.55 × 10^-6^ to 3.01 × 10^-1^ copies of ARG per copy of the 16S rRNA gene (Figure 1). The ARG *aadA*, which is associated with resistance to aminoglycosides, had the highest abundance ratio of 3.01 × 10^-1^ in the soil samples. Similarly, the tetracycline resistance gene *tet(B)*, which had the lowest abundance ratio of 1.55 × 10^-6^, was also found in the soil samples (Supplementary Table S3). For most of the samples, the abundance ratio range was between 10^-4^ and 10^-1^.

**FIGURE 2:**
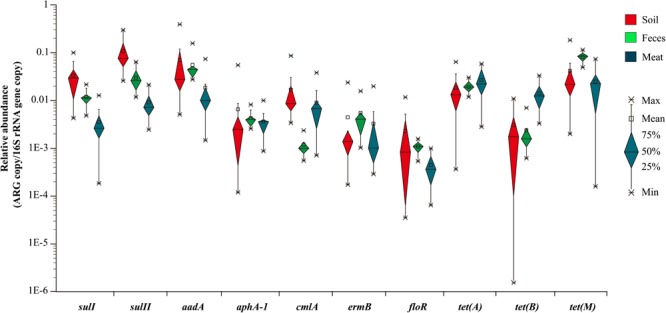
The relative average abundance of 10 ARGs in environmental and meat samples.

In general, the resistance genes *sulI, sulII, aadA, tet(A)*, and *tet(M)* had higher abundances than the other ARGs in the environmental samples (Figure 2). Moreover, the abundances of the resistance genes *sulII* and *aadA* were relatively high in the soil samples, with the average ratios of *sulII*/16S rRNA and *aadA*/16S rRNA reaching 1.08 × 10^-1^ and 7.0 × 10^-2^, respectively. In the fecal samples, abundances of *aadA* and *tet(M)* were much higher, with average ratios of 5.54 × 10^-2^ and 8.1 × 10^-2^, respectively. The abundances of all 10 ARGs detected in the fecal samples were in the order of: *tet(M)* > *aadA* > *sulII* > *tet(A)* > *sulI* > *ermB* > *aphA-1* > *tet(B)* > *cmlA* > *floR*. Compared with the environmental samples, the abundances of the detected ARGs in the meat samples were lower, except for those of *tet(A)* and *tet(B)*. Notably, the abundance of the tetracycline resistance gene *tet(B)* in the meat samples was much higher than in the environmental samples.

In summary, among the 10 representative ARGs, *sulII, aadA*, and *tet(M)*, which confer resistance to sulfanilamide, aminoglycosides, and tetracycline were the most abundant genes in both the environmental and meat samples, respectively. In contrast, the ARGs *aphA-1, ermB*, and *floR*, which are associated resistance to aminoglycosides, macrolides, and florfenicol had much lower abundances in most of the environmental and meat samples, respectively (Figure 2).

### Similarity Analysis of ARG Compositions

**FIGURE 3:**
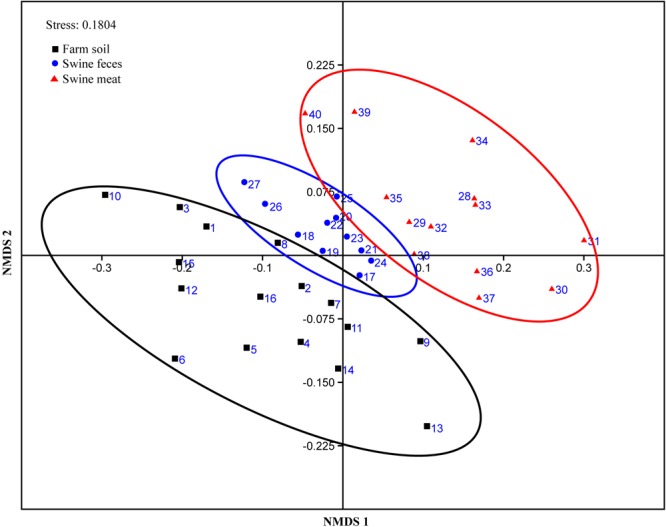
NMDS plot showing the ARG composition differences among the 40 representative environmental and meat samples.

The similarity of the ARG compositions in the 40 environmental and meat samples was evaluated using NMDS. Samples of the same type generally clustered more closely, which revealed that the grouping pattern was primarily influenced by sample type (Figure 3). For instance, the meat, feces, and most of the soil samples formed distinct clusters, especially the fecal samples which displayed high similarity in abundance (*p*-value = 0.96, evaluated by means of ANOVA statistical analysis). In addition, two fecal samples (F-10 and F-11) with the codes of 26 and 27, respectively, in the NMDS plot formed a cluster that was independent of the other fecal samples. Not surprisingly, these samples were collected in the same batch, which differed from those of the other fecal samples. Notably, among the three types of samples, the meat samples clustered more closely with the fecal samples, and this result was statistically supported by ANOVA analyses (*p*-value = 0.18).

### DGGE Analysis and Identification of DGGE Bands

**FIGURE 4:**
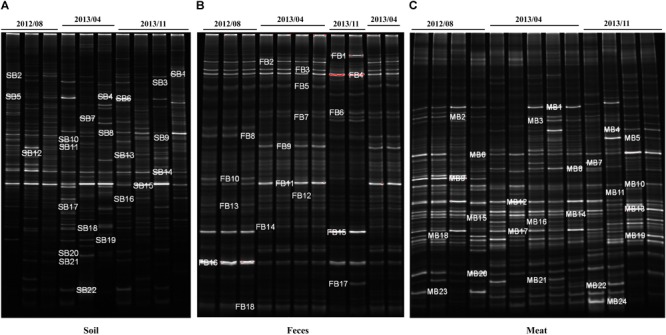
DGGE analysis of bacterial community composition in environmental and meat samples. **(A)** Farm soil; **(B)** Swine feces; **(C)** Swine meat. The denaturant gradient of the gels used for soil, feces, and meat samples were 45∼60%, 40∼55%, and 40∼60%, respectively.

The dominant bacterial community composition of the 40 soil, feces and meat samples (mentioned above) was analyzed with PCR-DGGE. The DGGE band patterns indicated complex dominant bacterial community composition across all sample types (Figure 4). Moreover, the band patterns of the soil samples displayed a higher degree of heterogeneity (Supplementary Figure S1a). In contrast, for the fecal samples, the composition of the bands in the DGGE profiles did not differ significantly among each other, especially among samples collected in the same batch (Supplementary Figure S1b). Compared with the soil samples, the band pattern of the meat samples indicated a lower bacterial diversity, but notably, the position and brightness of the bands among the meat samples was consistent (Supplementary Figure S1c), which indicated that the dominant bacteria in the meat samples were relatively stable.

To investigate the bacterial composition in the environmental and meat samples based on their PCR-DGGE bands, a total of 64 of the most frequent and obvious DGGE bands were marked and excised from the gels, and then purified and sequenced (Figure 4). As shown in Supplementary Table S4, 22, 18, and 24 bands were identified from the DGGE patterns for the soil, fecal, and meat samples, respectively. In the soil samples, among the 22 bands, 9 bands were identified as uncultured bacteria, which accounted for over 40% of the total bands. Moreover, the bacteria *Bacillus* sp. and *Clostridium* sp. were detected most frequently, both occupying three bands each. Similarly, in the fecal samples, 12 of a total 18 bands were identified as uncultured bacteria. Among the remaining six bands, two bands were identified as *Clostridium* spp., and two other bands were identified as *Arcobacter* spp. The last two bands were identified as a *Desulfovibrio* sp. and a *Tissierella* sp., respectively. In contrast to the soil and fecal samples, only one of the total 24 bands excised from the meat sample gel were identified as uncultured bacteria. The results showed that more than 14 species of known bacteria were identified from the meat sample gel, and four, three, three, two, and two bands were identified as *Serratia* spp., *Aeromonas* spp., *Pantoea* spp., *Enterobacter* spp., and *Bacillus* spp., respectively. Other species of bacteria, including common pathogens such as *Klebsiella pneumoniae*, were also identified from the meat samples (Supplementary Table S4).

### Phylogenetic Analysis

**FIGURE 5:**
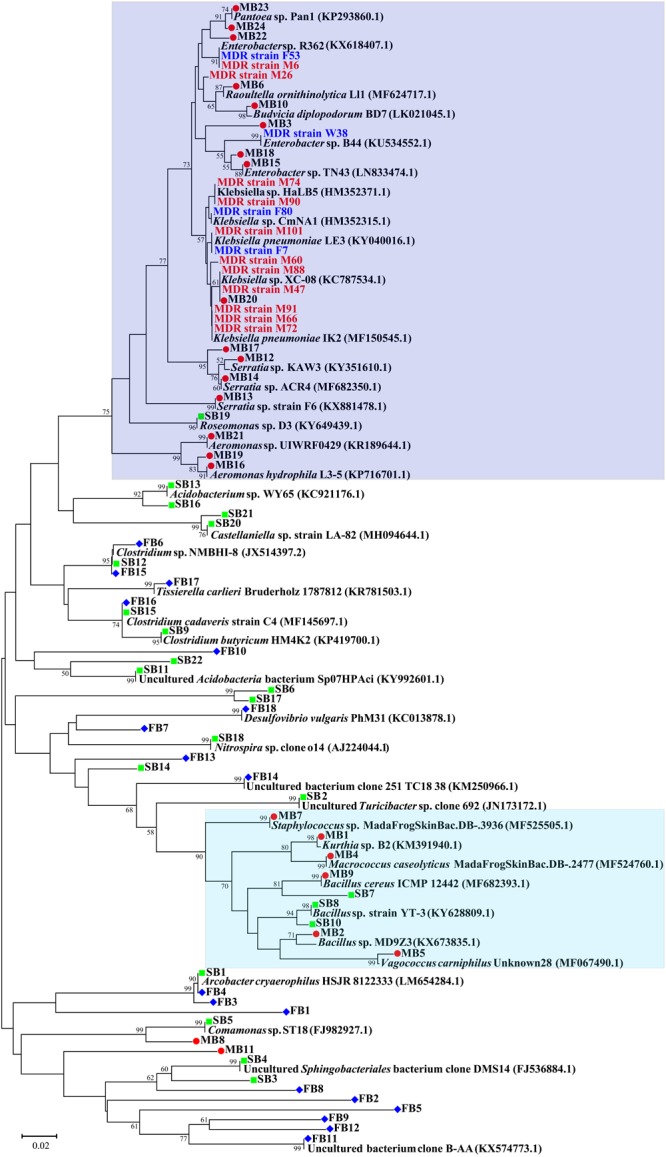
Neighbor-joining phylogenetic tree of the genes from DGGE of environmental and meat samples. MDR strains were isolated from the same farm in our previous study. M, pig meat; F, pig feces; and W, farm wastewater. The distinct clusters majorly formed by sequences from meat samples were highlighted with green and purple in background. Values on the branches represent the percentage of 1000 bootstrap replicates and bootstrap values over 50% are shown in the tree.

A neighbor-joining phylogenetic tree of the sequences of total 64 bands was constructed based on the maximum composite likelihood method. 17 of the 24 sequences of bands from the meat sample gel formed a very distinct and independent group, and showed high homology (Figure 5). Compared with the meat samples, the sequences of bands from the environmental sample gels showed relatively distant phylogenetic relationships. Furthermore, the bands MB12, MB13, MB14, and MB17 from the meat sample gel were identified as *Serratia* spp.; however, band MB13 was not found on the same branch of the phylogenetic tree as the other three bands. Band MB8, which was identified as *Comamonas* sp., shared one branch with band SB5 which was from the soil sample gel and shared 100% homology with *Comamonas* sp. ST18 (FJ982927.1). Moreover, bands FB4 and SB1 from the feces and soil sample gels shared 100% homology, respectively, and they were identified as *Arcobacter cryaerophilus*. Similarly, bands FB16 and SB15, which were identified as an uncultured *Clostridium* sp., also shared 100% homology. Five bands from the meat and soil sample gels were identified as *Bacillus* spp., though they shared relatively low homology. Generally, the sequences of the bands from the soil and fecal sample gels showed closer phylogenetic relationships. Notably, the band MB20 which was identified as *Klebsiella pneumoniae*, shared 100% homology with MDR strain M47 and M88 isolated from meat samples in the same pig farm.

## Discussion

Antibiotics have been commonly used in veterinary medicine worldwide for therapeutic use and to increase production in animal husbandry. Numerous previous studies have focused on analyzing the abundance of ARGs in pig farm environments (Heuer et al., 2008; Cheng et al., 2013; Zhu et al., 2013; Ma et al., 2015). Nevertheless, rarely studies were also detecting the relative abundance of ARGs in the produced pork meat and connecting it to the surrounding environment (feces and farm soil).

In this study, we analyzed samples from a large-scale pig farm, on which sulfamethoxazole, trimethoprim, tetracycline, gentamicin, streptomycin, chloramphenicol, florfenicol, and amoxicillin were widely used for the treatment of swine infections or as growth promoters. Sulfonamides/trimethoprim, tetracyclines, macrolides, penicillins and aminoglycosides are the most widely used groups of antibiotics in animal husbandry (Committee, 1999; Haller et al., 2002; Economou and Gousia, 2015), and consequently ARGs associated to these antibiotics are generally detected most frequently in various livestock farms (Ho et al., 2010; Cheng et al., 2013; Zhu et al., 2013; Tao et al., 2014). While for this study no exact amounts of the corresponding antibiotic doses administered were available, the high abundance of ARGs conferring resistance to these antibiotics is a good indicator that these antibiotics were consistently given on the farm.

In a previous study on this exact pig farm, we isolated 102 multidrug-resistant (MDR) enterobacterial strains, and identified MDR strains sharing 100% phylogenetic identity across the 3 different environments (meat, soil, and feces) (Liu et al., 2015). To further our understanding of the abundance and transfer of potentially antibiotic resistance bacteria on the pig farm we here moved from single isolates to a community wide detection of antibiotic resistance, as livestock farm environments are known to harbor a huge diversity of bacteria (McGarvey et al., 2004; Dowd et al., 2008). This approach involves detecting both, transmission of bacteria, as well as transfer of resistant genes from soil and fecal samples across the food production chain onto pork meat.

Since livestock farm environments harbored highly diverse bacteria (McGarvey et al., 2004; Dowd et al., 2008), the high throughput sequencing techniques could give much deeper insights into microbial community diversity compared with DGGE (Guo and Zhang, 2012). However, for the fresh meat samples, the PCR-DGGE technique remains a useful and economic tool to rapidly analyze the composition of dominant bacteria. In the past decade, the microbial diversity and main flora in fresh meat has been widely investigated using PCR-DGGE (Jiang et al., 2010, 2011; Osés et al., 2013; de Smidt, 2016; Koo et al., 2016). In this study, comparing with the soil and feces samples, the composition of bands from the meat samples showed high consistency across replicates, which indicated that dominant bacteria across meat samples were relatively stable. Unsurprisingly, the number of visible bands was lower than for both other sample types.

To investigate transfer of bacteria across environments we sequenced a total of 64 of the most frequent DGGE bands. Three bands from the meat sample were identified as *Enterobacter* sp. and *Klebsiella* sp., respectively, and bacteria of these two genera have previously been detected as the predominant MDR bacteria on the same pig farm (Liu et al., 2015). The detected bacteria of these groups showed close evolutionary relationship with the bacteria identified in this study (Figure 5), indicating that transfer of these MDR bacteria from the pig farm onto the meat might be occurring. Additionally, in the Bacilli group, species from soil as well as from meat samples are found in close proximity. Further, *Serratia, Aeromonas*, and *Pantoea* were identified as apparent on meat. All these bacteria are widely distributed in environmental and pork samples (Jiang and Shi, 2013; Greig et al., 2015; Roberts and Schwarz, 2016; Møretrø and Langsrud, 2017), and various ARGs have been detected in antibiotics resistant strains belonging to these bacterial genera (Batah et al., 2015; Liu et al., 2015; Le et al., 2016; Carnelli et al., 2017). Contrary, over 40% of the bands from the soil and fecal sample gels were identified as uncultured bacteria (Supplementary Table S4), and accounted for the vast majority of the total bacteria as expected from various environmental samples (Rappé and Giovannoni, 2003). Based on analysis of the created phylogenetic tree we can conclude that the composition of the predominant bacterial community in pork differed significantly from that in soil or fecal samples, however, we found several species that were closely related and potentially spread across the environments, including the previously isolated and highly medically relevant multi-drug resistant strain *Klebsiella pneumonia*, regularly involved in spreading ARGs from the environment to pathogens (Wyres and Holt, 2018).

There were more overlaps in bacterial community composition between meat and soil samples compared to meat and fecal samples. However, for the prevalence and composition of ARGs, a higher degree of similarity was detected among meat and fecal, rather than meat and soil samples. The prevalence of 26 ARGs in pork was surprisingly consistent with breeding environments, especially between the pork and feces. ARG composition of all 40 samples as detected using qPCR was subject to NMDS analysis using the Bray-Curtis distance. NMDS has been widely used in various environments to compare the bacterial communities of numerous samples (Guan et al., 2014; San Miguel et al., 2014; Xiong et al., 2015; Huo et al., 2017). But, it is also a useful tool to analyze the similarity of ARG compositions between different samples (Segawa et al., 2013; Li et al., 2015). Consistent with these previous studies, clustering in our study was mainly influenced by the sample origin. Further, among the three types of samples, the meat samples clustered more closely with the fecal samples (*p*-value = 0.18), combine the results mentioned above, strongly indicating that ARGs on meat samples can indeed originate from the fecal samples. This hypothesis can further be supported by the report that most bacterial genera detected on chilled pork are associated with fecal contamination during slaughtering (Zhao et al., 2015). And despite not detecting any immediate overlaps of sequenced DGGE bands between fecal and meat samples, identical MDR isolates found in both environments and mating experiments suggest that these bacteria furthermore harbor their resistance determinants on conjugative and thus self-transmissible plasmids that could spread to other bacteria on the meat (Liu et al., 2015).

Compared with fecal and meat samples, the soil samples did not only cluster further apart, but, consistent with the previously detected higher variance in bacterial composition also had a higher internal distance between replicates when analyzing the ARG content. The high abundance of ARGs in pig farm soils is generally assumed to primarily originate from the selection pressure of antibiotics originating from pig urine or feces (Tao et al., 2014). In this study, the soil sampling sites were widely distributed across the large-scale pig farm, therefore, the urine or feces pollution levels in soil samples did potentially differ substantially.

To complete this analysis the prevalence of 26 resistance genes was tested by amplification with commonly used ARG primers. Based on these results, the relative abundance of 10 representative ARGs, which were observed most frequently, was further detected with real-time PCR, allowing for a far more accurate and sensitive detection of ARGs than metagenomic sequencing analysis. To normalize the ARGs among the various samples, the relative abundance of the ARGs was expressed as copy of ARG per copy of 16S rRNA gene. The same calculation method has previously been used to estimate the overall bacterial abundance and to normalize ARGs to the bacterial population in samples from different sources (Gao et al., 2012; Cheng et al., 2013; Li et al., 2015; Subirats et al., 2017). The 10 tested ARGs, conferring resistance to six different classes of antibiotics, were detected with an abundance range between 3.01 × 10^-1^ and 1.55 × 10^-6^ per 16S rRNA copy in our samples.

Across all samples resistance to sulfanilamide (*sulI* and *sulII*), aminoglycoside (*aadA*) and tetracycline [*tet(A)* and *tet(M)*] were the most abundant ARGs. Based on information received from farm workers, these classes of antibiotics were consistently used in this large-scale pig farm. Consistent with our study, Cheng et al. (2013) reported detection of *sulI, sulII* and *tetM* with high relative abundance in livestock farms located in eastern China. Especially for sulfanilamide resistant genes, *sulI* and *sulII*, their relative abundance in this study was much higher than in other regions, such as United States (Munir and Xagoraraki, 2011) and Germany (Heuer et al., 2008), indicating a far increased and potential over-use of sulfanilamide antibiotics on our testing farm. Among our most frequently detected ARGs, *sulI* and *aadA*, are heavily associated with integron 1 gene cassettes (Binh et al., 2009; Byrne-Bailey et al., 2011; Liu et al., 2015), allowing their horizontal spread across communities and environments, and increasing their persistence as for example shown in manured soil (Zhang et al., 2015).

Tetracyclines are in general the most used antibiotics in pig farms, which are usually incorporated into animal feed to improve growth rate and feed efficiency (Sarmah et al., 2006). In this study, three tetracycline resistance genes [*tet(A), tet(B)*, and *tet(M)*] were part of real-time PCR analysis. All three of these tetracycline resistance genes have been observed frequently in various livestock farms (Cheng et al., 2013; Kyselková et al., 2015; Li et al., 2015; Ma et al., 2015). While *tet(A)* and *tet(M)* were detected with high abundance across all samples, *tet(B)* was detected with far increased frequencies on the meat samples.

The other 4 tested ARGs, *aphA-1, cmlA, ermB*, and *floR* conferring resistance to aminoglycosides, chloramphenicol, macrolides and florfenicol were found across all samples, but at relatively low frequencies. Among all tested ARGs, *floR* had the lowest average relative abundance in both environmental and meat samples, with the average ratios ranging from 2.3 × 10^-3^ to 4 × 10^-4^, consistent with a previous report (Li et al., 2015) where the relative abundance of *floR* in environmental samples from pig farms ranged from 2.02 × 10^-5^ to 1.33 × 10^-3^ copies/16S rRNA gene copies. ARG abundances were usually associated with the application of these antibiotics in livestock farms (Knapp et al., 2009; Gillings and Stokes, 2012; Zhu et al., 2013), therefore, the relatively low abundance of the above mentioned ARGs might be due to less use of the corresponding antibiotics on this pig farm.

## Conclusion

In conclusion, this study analyzed distribution and abundance of ARGs and dominate bacterial composition in environmental and pork samples from a large-scale pig farm where antibiotics were widely used. Our results demonstrated that there is a strong indication that ARGs and the associated MDR organisms potentially spread from the pig breeding environment to meat via the pork industry chain. These findings strongly indicate that the breeding environment is an important reservoir and breeding ground for antibiotic resistant bacteria and ARGs, which could be potentially transmitted to humans via the meat industry chain. Therefore, at the present time, the strategies for reasonable use of antibiotics, such as establishing regional management regimes for agricultural use of antibiotics, limiting the use of antibiotics as growth promoters and developing antibiotic substitutes, and establishment of scientific monitoring systems in animal husbandry are essential to limit the adverse effects of the abuse of antibiotics and to ensure the safety of animal-derived food and environment.

## Ethics Statement

The animals were processed according to the “Regulations for the administration of affairs concerning experimental animals” established by Guangdong Provincial Department of Science and Technology on the Use and Care of Animals. The experiments were approved by the Institutional Animal Care and Use Committee of Shenzhen University.

## Author Contributions

LS and ML designed the experiments and provided experimental materials. ZL and LY carried out experiments. ZL and UK analyzed sequencing data and wrote the manuscript.

## Conflict of Interest Statement

The authors declare that the research was conducted in the absence of any commercial or financial relationships that could be construed as a potential conflict of interest.

## Footnotes

1 http://www.ncbi.nlm.nih.gov/BLAST/
