# Supplementary material for: From Pig Breeding Environment to Subsequently Produced Pork: Comparative Analysis of Antibiotic Resistance Genes and Bacterial Community Composition
Source: Front Microbiol. 2019 Jan 29;10:43. doi: 10.3389/fmicb.2019.00043 (PMC6361818; doi:10.3389/fmicb.2019.00043)
Supplement: Supplementary file 1 [file Data_Sheet_1.docx]

**Frontiers in Microbiology**

Supplementary Material

**From pig breeding environment to subsequently produced pork: Comparative analysis of antibiotic resistance genes and bacterial community composition**

Zongbao Liu^1,2^, Uli Klümper^3,4^, Lei Shi^5^, Lei Ye^5*^, Meng Li^1*^

*1 Institute for Advanced Study, Shenzhen University, Shenzhen, Guangdong, P. R. China*

*2 Key Laboratory of Optoelectronic Devices and Systems of Ministry of Education and Guangdong Province, College of Optoelectronic Engineering, Shenzhen University, Shenzhen, Guangdong, P. R. China*

*3 ESI & CEC, Biosciences, University of Exeter, Penryn Campus, Cornwall, United Kingdom*

*4 European Centre for Environment and Human Health, University of Exeter, Truro, United Kingdom*

*5Institute of Food Safety and Nutrition, Jinan University, Guangzhou, Guangdong, P. R. China*

*Corresponding author:

Dr. Meng Li

Mailing address: Institute for Advanced Study, Shenzhen University, Shenzhen, Guangdong, P. R. China

Tel.:+86-755-26979250

E-mail Address: limeng848@szu.edu.cn

Dr. Lei Ye

Mailing address: Institute of Food Safety and Nutrition, Jinan University, Guangzhou, Guangdong, P. R. China

Tel: + 86-20-85220217

E-mail: [176003871@qq.com](mailto:176003871@qq.com)

Table S1 PCR primers used in this study (Ng et al., 2001; Van et al., 2008; Lin et al., 2012; Jiang and Shi, 2013; Yamamoto et al., 2013; Lv et al., 2014; Mazurek et al., 2014)

| Antimicrobial resistance | | Gene | | Sequence (5'-3') | Length (bp) | Annealing temperature | |
| --- | --- | --- | --- | --- | --- | --- | --- |
| V3 region | | 338F | | CCTACGGGAGGCAGCAG | 194 | 55℃ | |
|  | | 518R | | ATTACCGCGGCTGCTGG |  |  | |
| Sulfonamide | | *sulI* | | TTCGGCATTCTGAATCTCAC | 822 | 58℃ | |
|  | |  | | ATGATCTAACCCTCGGTCTC |  |  | |
|  | | *sulII* | | CGGCATCGTCAACATAACC | 722 | 60℃ | |
|  | |  | | GTGTGCGGATGAAGTCAG |  |  | |
| Trimethoprim | | *dhfrV* | | CTGCAAAAGCGAAAAACGG | 432 | 58℃ | |
|  | |  | | AGCAATAGTTAATGTTTGAGCTAAAG |  |  | |
|  | | *dhfrI* | | AAGAATGGAGTTATCGGGAATG | 391 | 58℃ | |
|  | |  | | GGGTAAAAACTGGCCTAAAATTG |  |  | |
|  | | *DfrA12* | | GCCGTGGGTCGATGTTTGAT | 395 | 55℃ | |
|  | |  | | TTCACCACCACCAGACACA |  |  | |
|  | | *df rA17* | | GTTAGCCTTTTTTCCAAATCTGGTATG | 475 | 55℃ | |
|  | |  | | TTGAAAATATTATTGATTTCTGCAGTG |  |  | |
| Aminoglycoside | | *aadA* | | TGATTTGCTGGTTACGGTGAC | 284 | 58℃ | |
|  | |  | | CGCTATGTTCTCTTGCTTTTG |  |  | |
|  | | *aadB* | | TCCAGAACCTTGACCGAAC | 700 | 57℃ | |
|  | |  | | GCAAGACCTCAACCTTTTCC |  |  | |
|  | | *aac(3)-I* | | ACCTACTCCCAACATCAGCC | 169 | 58℃ | |
|  | |  | | ATATAGATCTCACTACGCGC |  |  | |
|  | | *aphA-1* | | ATGGGCTCGCGATAATGTC | 600 | 58℃ | |
|  | |  | | CTCACCGAGGCAGTTCCAT |  |  | |
|  | | *aac(3)-IV* | | CTTCAGGATGGCAAGTTGGT | 286 | 58℃ | |
|  | |  | | TCATCTCGTTCTCCGCTCAT |  |  | |
| Chloramphenicol | | *catI* | | AGTTGCTCAATGTACCTATAACC | 547 | 58℃ | |
|  | |  | | TTGTAATTCATTAAGCATTCTGCC |  |  | |
|  | *cmlA* | | CCGCCACGGTGTTGTTGTTATC | | 698 | 58℃ |  |
|  |  | | CACCTTGCCTGCCCATCATTAG | |  |  |  |
| Beta-lactam | *blaSHV* | | TCGCCTGTGTATTATCTCCC | | 768 | 58℃ |  |
|  |  | | CGCAGATAAATCACCACAATG | |  |  |  |
|  | *blaOXA* | | GCAGCGCCAGTGCATCAAC | | 198 | 58℃ |  |
|  |  | | CCGCATCAAATGCCATAAGTG | |  |  |  |
|  | *blaTEM* | | GAGTATTCAACATTTTCGT | | 857 | 58℃ |  |
|  |  | | ACCAATGCTTAATCAGTGA | |  |  |  |
| AmpC's | *citM* | | TGGCCAGAACTGACAGGCAAA | | 462 | 58℃ |  |
|  |  | | TTTCTCCTGAACGTGGCTGGC | |  |  |  |
|  | *moxM* | | GCTGCTCAAGGAGCACAGGAT | | 520 | 58℃ |  |
|  |  | | CACATTGACATAGGTGTGGTGC | |  |  |  |
|  | *dhaM* | | AACTTTCACAGGTGTGCTGGGT | | 405 | 58℃ |  |
|  |  | | CCGTACGCATACTGGCTTTGC | |  |  |  |
| Macrolide | *ereA* | | GCCGGTGCTCATGAACTTGAG | | 419 | 58℃ |  |
|  |  | | CGACTCTATTCGATCAGAGGC | |  |  |  |
|  | *ermB* | | AGTAACGGTACTTAAATTGTTTAC | | 639 | 52℃ |  |
|  |  | | GAAAAGGTACTCAACCAAATA | |  |  |  |
| Florfenicol | *floR* | | TATCTCCCTGTCGTTCCAG | | 399 | 58℃ |  |
|  |  | | AGAACTCGCCGATCAATG | |  |  |  |
| Tetracycline | *tet(A)* | | GCTACATCCTGCTTGCCTTC | | 210 | 55℃ |  |
|  |  | | CATAGATCGCCGTGAAGAGG | |  |  |  |
|  | *tet(B)* | | TTGGTTAGGGGCAAGTTTTG | | 659 | 55℃ |  |
|  |  | | GTAATGGGCCAATAACACCG | |  |  |  |
|  | *tet(M)* | | GTGGACAAAGGTACAACGAG | | 406 | 55℃ |  |
|  |  | | CGGTAAAGTTCGTCACACAC | |  |  |  |
|  | *tet(D)* | | AAACCATTACGGCATTCTGC | | 787 | 55℃ |  |
|  |  | | GACCGGATACACCATCCATC | |  |  |  |

Table S2 Presence of antibiotic resistance genes in environmental and meat samples

| Samples | Sampling time | SA | | TMP | | | | Aminoglycoside | | | | | CHL | | Beta-lactam | | | AmpC's | | | MAC | | FFC | TET | | | |
| --- | --- | --- | --- | --- | --- | --- | --- | --- | --- | --- | --- | --- | --- | --- | --- | --- | --- | --- | --- | --- | --- | --- | --- | --- | --- | --- | --- |
|  |  | *sulI* | *sulII* | *dhfrV* | *dhfrI* | *DfrA12* | *df rA17* | *aadA* | *aadB* | *aac(3)-I* | *aphA-1* | *aac(3)-IV* | *catI* | *cmlA* | *blaSHV* | *blaOXA* | *blaTEM* | *citM* | *moxM* | *dhaM* | *ereA* | *ermB* | *floR* | *tet(A)* | *tet(B)* | *tet(M)* | *tet(D)* |
| Farm soil | |  |  |  |  |  |  |  |  |  |  |  |  |  |  |  |  |  |  |  |  |  |  |  |  |  |  |
| S-1 | 2012/08 | + | + | - | - | - | - | + | - | - | + | - | - | + | - | - | - | - | - | - | - | + | + | + | - | + | - |
| S-2 | 2012/08 | + | + | - | - | - | - | + | - | - | + | - | - | + | - | - | - | - | - | - | - | + | + | + | + | + | - |
| S-3 | 2012/08 | + | + | - | - | - | - | + | - | - | + | - | - | + | - | - | - | - | - | - | - | + | + | + | + | + | - |
| S-4 | 2012/08 | + | + | - | - | - | - | + | - | - | + | - | - | + | - | - | - | - | - | - | - | - | + | + | + | + | - |
| S-5 | 2012/08 | + | + | - | - | - | - | + | - | - | + | - | - | + | - | - | - | - | - | - | - | + | + | + | + | + | - |
| S-6 | 2012/08 | + | + | - | - | - | - | + | - | - | + | - | - | + | - | - | - | - | - | - | - | + | + | + | + | + | - |
| S-7 | 2013/04 | + | + | - | - | - | - | + | - | - | + | - | - | + | - | - | - | - | - | - | - | + | + | + | - | + | - |
| S-8 | 2013/04 | + | + | - | - | - | - | + | - | - | + | - | - | + | - | - | - | - | - | - | - | + | + | + | + | + | - |
| S-9 | 2013/04 | + | + | - | - | - | - | + | - | - | + | - | - | + | - | - | - | - | - | - | - | + | + | + | + | + | - |
| S-10 | 2013/04 | + | + | - | - | - | + | + | - | - | + | - | - | + | - | - | - | - | - | - | - | + | + | + | + | + | - |
| S-11 | 2013/04 | + | + | - | - | - | - | + | - | - | + | - | - | + | - | - | - | - | - | - | - | + | + | + | + | + | - |
| S-12 | 2013/04 | + | + | - | - | - | - | + | - | - | + | - | - | + | - | - | - | - | - | - | - | + | + | + | - | + | - |
| S-13 | 2013/04 | + | + | - | - | - | - | + | - | - | + | - | - | + | - | - | - | - | - | - | - | + | + | + | - | + | - |
| S-14 | 2013/04 | + | + | - | - | - | - | + | - | - | + | - | - | + | - | - | - | - | - | - | - | + | + | + | - | + | - |
| S-15 | 2013/04 | + | + | - | - | - | + | + | - | - | + | - | - | + | - | - | - | - | - | - | - | + | + | - | - | + | - |
| S-16 | 2013/04 | + | + | - | - | - | - | + | - | - | + | - | - | + | - | - | - | - | - | - | - | + | + | + | - | + | - |
| S-17 | 2013/04 | + | + | - | - | - | - | + | - | - | + | - | - | + | - | - | - | - | - | - | - | + | + | + | - | + | - |
| S-18 | 2013/04 | + | + | - | - | - | - | + | - | - | + | - | - | + | - | - | - | - | - | - | - | + | + | + | + | + | - |
| S-19 | 2013/11 | + | + | - | - | - | + | + | + | - | + | - | - | + | - | - | - | - | - | - | - | + | + | + | + | + | - |
| S-20 | 2013/11 | + | + | - | - | - | + | + | + | - | + | - | - | + | - | - | - | - | - | - | - | + | + | - | + | + | - |
| S-21 | 2013/11 | + | + | - | - | - | + | + | + | - | + | - | - | + | - | - | - | - | - | - | - | + | + | + | - | + | - |
| S-22 | 2013/11 | + | + | - | - | - | - | + | + | - | + | - | - | + | - | - | - | - | - | - | - | - | + | + | - | + | - |
| S-23 | 2013/11 | + | + | - | - | - | - | + | - | - | + | - | - | + | - | - | - | - | - | - | - | - | + | - | - | + | - |
| S-24 | 2013/11 | + | + | - | - | - | - | + | - | - | + | - | - | + | - | - | - | - | - | - | - | - | + | + | - | + | - |
| S-25 | 2013/11 | + | + | - | - | - | - | + | - | - | + | - | - | + | - | - | - | - | - | - | - | + | + | + | - | + | - |
| S-26 | 2013/11 | + | + | - | - | - | + | + | + | - | + | - | - | + | - | - | - | - | - | - | - | + | + | + | + | + | - |
| S-27 | 2013/11 | + | + | - | - | - | + | + | + | - | + | - | - | + | - | - | - | - | - | - | - | + | + | + | + | + | - |
| Swine feces | |  |  |  |  |  |  |  |  |  |  |  |  |  |  |  |  |  |  |  |  |  |  |  |  |  |  |
| F-1 | 2012/08 | + | + | - | - | - | + | + | - | - | + | - | - | + | - | - | - | - | - | - | - | + | + | + | + | + | - |
| F-2 | 2012/08 | + | + | - | - | - | + | + | - | - | + | - | - | + | - | - | - | - | - | - | - | + | + | + | + | + | - |
| F-3 | 2012/08 | + | + | - | - | - | + | + | - | - | + | - | - | + | - | - | - | - | - | - | - | + | + | + | + | + | - |
| F-4 | 2013/04 | + | + | - | - | + | + | + | - | - | + | - | - | + | - | - | - | - | - | - | - | + | + | + | + | + | - |
| F-5 | 2013/04 | + | + | - | - | + | + | + | - | - | + | - | - | + | - | - | - | - | - | - | - | + | + | + | + | + | - |
| F-6 | 2013/04 | + | + | - | - | + | + | + | - | - | + | - | - | + | - | - | - | - | - | - | - | + | + | + | + | + | - |
| F-7 | 2013/04 | + | + | - | - | + | + | + | - | - | + | - | - | + | - | - | - | - | - | - | - | + | + | + | + | + | - |
| F-8 | 2013/04 | + | + | - | - | + | + | + | - | - | + | - | - | + | - | - | - | - | - | - | - | + | + | + | + | + | - |
| F-9 | 2013/04 | + | + | - | - | + | + | + | - | - | + | - | - | + | - | - | - | - | - | - | - | + | + | + | + | + | - |
| F-10 | 2013/11 | + | + | - | - | - | + | + | - | - | + | - | - | + | - | - | - | - | - | - | - | + | + | + | + | + | - |
| F-11 | 2013/11 | + | + | - | - | - | + | + | - | - | + | - | - | + | - | - | - | - | - | - | - | + | + | + | + | + | - |
| F-12 | 2013/11 | + | + | - | - | - | + | + | - | - | + | - | - | + | - | - | - | - | - | - | - | + | + | + | + | + | - |
| F-13 | 2013/11 | + | + | - | - | - | + | + | - | - | + | - | - | + | - | - | - | - | - | - | - | + | + | + | + | + | - |
| F-14 | 2013/11 | + | + | - | - | - | + | + | - | - | + | - | - | + | - | - | - | - | - | - | - | - | + | + | + | + | - |
| F-15 | 2013/11 | + | + | - | - | - | + | + | - | - | + | - | - | + | - | - | - | - | - | - | - | + | + | + | + | + | - |
| F-16 | 2013/11 | + | + | - | - | - | + | + | - | - | + | - | - | + | - | - | - | - | - | - | - | + | + | + | + | + | - |
| F-17 | 2013/11 | + | + | - | - | - | - | + | - | - | + | - | - | + | - | - | - | - | - | - | - | + | + | + | + | + | - |
| F-18 | 2013/11 | + | + | - | - | - | + | + | - | - | + | - | - | + | - | - | - | - | - | - | - | + | + | - | + | + | - |
| F-19 | 2013/11 | + | + | - | - | - | + | + | - | - | + | - | - | + | - | - | - | - | - | - | - | + | + | + | + | + | - |
| Swine meat | |  |  |  |  |  |  |  |  |  |  |  |  |  |  |  |  |  |  |  |  |  |  |  |  |  |  |
| M-1 | 2012/08 | + | + | - | - | - | - | + | - | - | + | - | - | + | - | - | - | - | - | - | - | + | + | + | + | + | - |
| M-2 | 2012/08 | + | + | - | - | - | - | + | - | - | + | - | - | + | - | - | - | - | - | - | - | + | + | + | + | + | - |
| M-3 | 2012/08 | + | + | - | - | - | - | + | - | - | + | - | - | + | - | - | - | - | - | - | - | + | + | + | + | + | - |
| M-4 | 2012/08 | + | + | - | - | - | - | + | - | - | + | - | - | + | - | - | - | - | - | - | - | + | + | + | + | + | - |
| M-5 | 2012/08 | + | + | - | - | - | - | + | - | - | + | - | - | + | - | - | - | - | - | - | - | + | + | + | + | + | - |
| M-6 | 2012/08 | + | + | - | - | - | - | + | - | - | + | - | - | + | - | - | - | - | - | - | - | + | + | + | + | + | - |
| M-7 | 2012/08 | + | + | - | - | - | + | + | - | - | + | - | - | + | - | - | - | - | - | - | - | + | + | + | + | + | - |
| M-8 | 2013/04 | + | + | - | - | - | - | + | - | - | + | - | - | + | - | - | - | - | - | - | - | + | + | + | + | - | - |
| M-9 | 2013/04 | + | + | - | - | - | - | + | - | - | + | - | - | + | - | - | - | - | - | - | - | + | + | + | + | + | - |
| M-10 | 2013/04 | + | + | - | - | - | - | + | - | - | + | - | - | + | - | - | - | - | - | - | - | + | + | + | + | + | - |
| M-11 | 2013/04 | + | + | - | - | - | + | + | - | - | + | - | - | + | - | - | - | - | - | - | - | + | + | + | + | - | - |
| M-12 | 2013/04 | + | + | - | - | - | + | + | - | - | + | - | - | + | - | - | - | - | - | - | - | + | + | + | + | - | - |
| M-13 | 2013/04 | + | + | - | - | - | + | + | - | - | + | - | - | + | - | - | - | - | - | - | - | + | + | + | + | - | - |
| M-14 | 2013/04 | + | + | - | - | - | + | + | - | - | + | - | - | + | - | - | - | - | - | - | - | + | + | + | + | + | - |
| M-15 | 2013/04 | + | + | - | - | - | + | + | - | - | + | - | - | + | - | - | - | - | - | - | - | + | + | + | + | + | - |
| M-16 | 2013/11 | + | + | - | - | - | - | + | - | - | + | - | - | + | - | - | - | - | - | - | - | + | + | + | + | - | - |
| M-17 | 2013/11 | + | + | - | - | - | + | + | - | - | + | - | - | + | - | - | - | - | - | - | - | + | + | + | + | + | - |
| M-18 | 2013/11 | + | + | - | - | + | + | + | - | - | + | - | - | + | - | - | - | - | - | - | - | + | + | + | + | + | - |
| M-19 | 2013/11 | + | + | - | - | + | + | + | - | - | + | - | - | + | - | - | - | - | - | - | - | + | + | + | + | + | - |
| M-20 | 2013/11 | + | + | - | - | + | + | + | - | - | + | - | - | + | - | - | - | - | - | - | - | + | + | + | + | + | - |
| M-21 | 2013/11 | + | + | - | - | + | + | + | - | - | + | - | - | + | - | - | - | - | - | - | - | + | + | + | + | + | - |
| M-22 | 2013/11 | + | + | - | - | + | + | + | - | - | + | - | - | + | - | - | - | - | - | - | - | + | + | + | + | + | - |

SA:Sulfonamide ; TMP:Trimethoprim; CHL: Chloramphenicol; MAC: Macrolide; FFC: Florfenicol; TET: Tetracycline

“+” and “-” represents positive and negative PCR results, respectively

Table S3 Copies ratio of 10 antibiotic resistant genes and V3 region in samples

|  | *sulI*/V3 | *sulII*/V3 | *aadA*/V3 | *aphA-1*/V3 | *cmlA*/V3 | *ermB*/V3 | *floR*/V3 | *tet(A)* /V3 | *tet(B)* /V3 | *tet(M)* /V3 |
| --- | --- | --- | --- | --- | --- | --- | --- | --- | --- | --- |
| Farm soil | |  |  |  |  |  |  |  |  |  |
| S-2 | 6.57×10^-2^ | 1.58×10^-1^ | 1.08×10^-1^ | 2.41×10^-3^ | 2.06×10^-2^ | 9.25×10^-3^ | 8.30×10^-4^ | 3.61×10^-2^ | 4.48×10^-3^ | 1.24×10^-1^ |
| S-3 | 3.53×10^-2^ | 7.57×10^-2^ | 2.74×10^-2^ | 2.60×10^-4^ | 6.02×10^-3^ | 8.12×10^-4^ | 6.23×10^-5^ | 2.43×10^-2^ | 4.98×10^-4^ | 5.44×10^-2^ |
| S-5 | 1.00×10^-1^ | 1.60×10^-1^ | 1.13×10^-1^ | 5.75×10^-3^ | 2.94×10^-2^ | 2.36×10^-2^ | 3.89×10^-3^ | 3.51×10^-2^ | 7.58×10^-3^ | 1.83×10^-1^ |
| S-8 | 3.43×10^-2^ | 6.82×10^-2^ | 2.38×10^-2^ | 4.31×10^-4^ | 5.63×10^-3^ | 9.88×10^-4^ | 3.53×10^-5^ | 6.01×10^-3^ | 7.40×10^-4^ | 2.18×10^-2^ |
| S-10 | 1.06×10^-2^ | 1.70×10^-1^ | 8.62×10^-3^ | 4.57×10^-3^ | 4.32×10^-3^ | 7.10×10^-4^ | 4.25×10^-5^ | 1.41×10^-2^ | 2.18×10^-3^ | 3.85×10^-2^ |
| S-11 | 1.23×10^-2^ | 2.97×10^-1^ | 4.09×10^-2^ | 2.77×10^-4^ | 1.44×10^-2^ | 1.74×10^-4^ | 6.78×10^-5^ | 1.31×10^-2^ | 1.74×10^-3^ | 1.85×10^-2^ |
| S-14 | 2.95×10^-2^ | 5.05×10^-2^ | 2.05×10^-2^ | 6.70×10^-4^ | 8.56×10^-3^ | 1.78×10^-3^ | 1.55×10^-4^ | 1.13×10^-2^ | 4.21×10^-6^ | 4.49×10^-2^ |
| S-15 | 5.47×10^-2^ | 5.75×10^-2^ | 5.75×10^-2^ | 1.41×10^-2^ | 2.03×10^-2^ | 2.10×10^-3^ | 2.54×10^-3^ | 3.23×10^-2^ | 8.25×10^-6^ | 5.99×10^-2^ |
| S-16 | 5.77×10^-3^ | 2.57×10^-2^ | 5.10×10^-3^ | 1.20×10^-4^ | 3.39×10^-3^ | 8.87×10^-4^ | 4.01×10^-5^ | 3.44×10^-3^ | 1.55×10^-6^ | 3.41×10^-2^ |
| S-18 | 6.00×10^-2^ | 1.79×10^-1^ | 3.01×10^-1^ | 5.46×10^-2^ | 8.59×10^-2^ | 1.35×10^-2^ | 1.17×10^-2^ | 6.39×10^-2^ | 1.09×10^-2^ | 3.85×10^-2^ |
| S-19 | 1.11×10^-2^ | 4.84×10^-2^ | 2.16×10^-2^ | 1.27×10^-3^ | 9.35×10^-3^ | 4.72×10^-4^ | 1.95×10^-4^ | 1.44×10^-2^ | 2.28×10^-3^ | 1.28×10^-2^ |
| S-20 | 3.89×10^-2^ | 1.10×10^-1^ | 1.12×10^-1^ | 3.05×10^-3^ | 6.71×10^-3^ | 1.39×10^-3^ | 5.21×10^-3^ | 3.66×10^-4^ | 2.55×10^-3^ | 4.55×10^-3^ |
| S-21 | 5.38×10^-3^ | 2.69×10^-2^ | 5.12×10^-3^ | 2.48×10^-3^ | 6.75×10^-3^ | 3.98×10^-4^ | 9.12×10^-4^ | 2.25×10^-3^ | 9.88×10^-5^ | 2.39×10^-3^ |
| S-25 | 4.28×10^-3^ | 6.03×10^-2^ | 1.58×10^-2^ | 2.71×10^-3^ | 9.33×10^-3^ | 1.38×10^-3^ | 4.09×10^-3^ | 6.71×10^-3^ | 1.77×10^-4^ | 1.26×10^-2^ |
| S-26 | 4.39×10^-2^ | 1.58×10^-1^ | 1.19×10^-1^ | 8.64×10^-3^ | 3.01×10^-2^ | 1.13×10^-2^ | 4.80×10^-3^ | 1.32×10^-2^ | 8.55×10^-3^ | 1.23×10^-2^ |
| S-27 | 1.94×10^-2^ | 8.23×10^-2^ | 5.17×10^-2^ | 2.78×10^-3^ | 6.84×10^-3^ | 2.30×10^-3^ | 2.36×10^-3^ | 1.76×10^-2^ | 7.26×10^-3^ | 2.01×10^-3^ |
| Swine feces | |  |  |  |  |  |  |  |  |  |
| F-1 | 1.29×10^-2^ | 2.60×10^-2^ | 3.01×10^-2^ | 2.56×10^-3^ | 1.00×10^-3^ | 1.62×10^-3^ | 5.37×10^-4^ | 1.21×10^-2^ | 1.02×10^-3^ | 5.43×10^-2^ |
| F-2 | 2.15×10^-2^ | 4.21×10^-2^ | 5.13×10^-2^ | 8.17×10^-3^ | 5.50×10^-4^ | 1.87×10^-3^ | 1.45×10^-3^ | 2.02×10^-2^ | 2.60×10^-3^ | 1.15×10^-1^ |
| F-3 | 1.80×10^-2^ | 3.46×10^-2^ | 4.80×10^-2^ | 6.32×10^-3^ | 2.36×10^-3^ | 1.03×10^-3^ | 1.34×10^-3^ | 1.77×10^-2^ | 2.05×10^-3^ | 7.40×10^-2^ |
| F-4 | 1.30×10^-2^ | 1.87×10^-2^ | 3.90×10^-2^ | 4.51×10^-3^ | 8.95×10^-4^ | 4.04×10^-3^ | 8.02×10^-4^ | 2.48×10^-2^ | 1.46×10^-3^ | 9.63×10^-2^ |
| F-5 | 1.13×10^-2^ | 1.65×10^-2^ | 3.01×10^-2^ | 3.30×10^-3^ | 8.08×10^-4^ | 4.44×10^-3^ | 1.01×10^-3^ | 1.52×10^-2^ | 1.07×10^-3^ | 6.85×10^-2^ |
| F-6 | 9.21×10^-3^ | 2.77×10^-2^ | 4.45×10^-2^ | 4.60×10^-3^ | 1.26×10^-3^ | 4.63×10^-3^ | 1.14×10^-3^ | 2.48×10^-2^ | 1.57×10^-3^ | 9.93×10^-2^ |
| F-7 | 1.09×10^-2^ | 1.98×10^-2^ | 3.45×10^-2^ | 3.58×10^-3^ | 7.74×10^-4^ | 3.69×10^-3^ | 1.09×10^-3^ | 1.92×10^-2^ | 1.18×10^-3^ | 8.39×10^-2^ |
| F-8 | 8.51×10^-3^ | 1.61×10^-2^ | 2.75×10^-2^ | 2.60×10^-3^ | 6.34×10^-4^ | 4.05×10^-3^ | 8.17×10^-4^ | 1.19×10^-2^ | 6.19×10^-4^ | 6.58×10^-2^ |
| F-9 | 8.66×10^-3^ | 1.18×10^-2^ | 4.63×10^-2^ | 3.98×10^-3^ | 1.19×10^-3^ | 5.51×10^-3^ | 1.07×10^-3^ | 3.01×10^-2^ | 1.74×10^-3^ | 9.81×10^-2^ |
| F-10 | 4.82×10^-3^ | 6.42×10^-2^ | 1.02×10^-1^ | 2.86×10^-3^ | 1.34×10^-3^ | 1.57×10^-2^ | 1.11×10^-3^ | 1.91×10^-2^ | 7.02×10^-3^ | 8.69×10^-2^ |
| F-11 | 1.18×10^-2^ | 5.24×10^-2^ | 1.56×10^-1^ | 4.29×10^-3^ | 2.27×10^-3^ | 1.33×10^-2^ | 1.58×10^-3^ | 1.61×10^-2^ | 5.71×10^-3^ | 4.91×10^-2^ |
| Swine meat | |  |  |  |  |  |  |  |  |  |
| M-1 | 1.62×10^-3^ | 6.42×10^-3^ | 8.84×10^-3^ | 1.92×10^-3^ | 2.56×10^-3^ | 3.11×10^-4^ | 4.93×10^-4^ | 4.63×10^-2^ | 9.65×10^-3^ | 2.24×10^-2^ |
| M-2 | 2.28×10^-3^ | 2.13×10^-2^ | 1.58×10^-2^ | 3.78×10^-3^ | 6.78×10^-3^ | 4.88×10^-4^ | 7.91×10^-4^ | 5.84×10^-2^ | 7.80×10^-3^ | 3.43×10^-2^ |
| M-4 | 4.44×10^-4^ | 6.00×10^-3^ | 4.05×10^-3^ | 1.22×10^-3^ | 1.32×10^-3^ | 2.89×10^-4^ | 1.15×10^-4^ | 2.83×10^-3^ | 3.29×10^-3^ | 1.46×10^-2^ |
| M-6 | 1.84×10^-4^ | 2.46×10^-3^ | 2.54×10^-3^ | 8.75×10^-4^ | 7.13×10^-4^ | 3.58×10^-4^ | 6.49×10^-5^ | 1.31×10^-2^ | 4.34×10^-3^ | 5.28×10^-3^ |
| M-9 | 5.03×10^-3^ | 4.82×10^-3^ | 2.19×10^-2^ | 3.87×10^-3^ | 6.87×10^-3^ | 8.72×10^-4^ | 1.71×10^-4^ | 2.22×10^-2^ | 1.53×10^-2^ | 2.49×10^-2^ |
| M-10 | 3.39×10^-3^ | 4.47×10^-3^ | 6.37×10^-3^ | 3.85×10^-3^ | 7.41×10^-3^ | 5.99×10^-4^ | 3.64×10^-4^ | 3.29×10^-2^ | 1.45×10^-2^ | 2.05×10^-2^ |
| M-11 | 2.62×10^-3^ | 1.24×10^-2^ | 1.02×10^-2^ | 2.98×10^-3^ | 9.70×10^-3^ | 5.86×10^-3^ | 9.94×10^-4^ | 1.52×10^-2^ | 2.42×10^-2^ | 1.59×10^-4^ |
| M-14 | 4.77×10^-3^ | 1.65×10^-2^ | 1.85×10^-2^ | 5.33×10^-3^ | 1.55×10^-2^ | 1.98×10^-2^ | 2.34×10^-4^ | 2.92×10^-2^ | 3.30×10^-2^ | 4.85×10^-2^ |
| M-15 | 2.47×10^-3^ | 1.01×10^-2^ | 6.08×10^-3^ | 2.02×10^-3^ | 5.72×10^-3^ | 1.01×10^-3^ | 1.13×10^-4^ | 9.94×10^-3^ | 1.24×10^-2^ | 2.42×10^-2^ |
| M-17 | 1.57×10^-3^ | 2.50×10^-3^ | 1.47×10^-3^ | 3.61×10^-3^ | 1.25×10^-3^ | 2.17×10^-3^ | 6.81×10^-4^ | 4.31×10^-3^ | 6.71×10^-3^ | 4.88×10^-2^ |
| M-18 | 2.72×10^-3^ | 7.69×10^-3^ | 1.00×10^-2^ | 3.62×10^-3^ | 4.71×10^-3^ | 2.00×10^-3^ | 2.26×10^-4^ | 1.35×10^-2^ | 4.93×10^-3^ | 7.33×10^-2^ |
| M-19 | 6.45×10^-3^ | 7.14×10^-3^ | 6.15×10^-2^ | 3.91×10^-3^ | 1.62×10^-2^ | 3.53×10^-3^ | 6.39×10^-4^ | 4.47×10^-2^ | 1.98×10^-2^ | 1.37×10^-3^ |
| M-22 | 1.27×10^-2^ | 1.63×10^-2^ | 7.33×10^-2^ | 9.97×10^-3^ | 3.80×10^-2^ | 5.14×10^-3^ | 9.52×10^-4^ | 5.65×10^-2^ | 2.87×10^-2^ | 3.80×10^-3^ |

Table S4 Identification of the genes from DGGE of environmental and meat samples

| Swine meat | | Farm soil | | Swine feces | |
| --- | --- | --- | --- | --- | --- |
| Bands | Genus | Bands | Genus | Bands | Genus |
| MB1 | *Kurthia* sp. | SB1 | *Arcobacter cryaerophilus* | FB1 | Uncultured bacterium |
| MB2 | *Bacillus* sp. | SB2 | Uncultured *Turicibacter* sp. | FB2 | Uncultured bacterium |
| MB3 | *Morganella* sp. | SB3 | *Ferruginibacter* sp. | FB3 | *Arcobacter* sp. F61 |
| MB4 | *Macrococcus caseolyticus* | SB4 | Uncultured Sphingobacteriales bacterium | FB4 | *Arcobacter cryaerophilus* |
| MB5 | *Vagococcus* sp. | SB5 | *Comamonas* sp. | FB5 | Uncultured bacterium |
| MB6 | *Raoultella ornithinolytica* | SB6 | Uncultured bacterium | FB6 | *Clostridium* sp. |
| MB7 | *Staphylococcus* sp. | SB7 | *Bacillus* sp. | FB7 | Uncultured *Clostridium* sp. |
| MB8 | *Comamonas* sp. | SB8 | *Bacillus* sp. | FB8 | Uncultured bacterium |
| MB9 | *Bacillus* sp. | SB9 | *Clostridium* sp. | FB9 | Uncultured bacterium |
| MB10 | *Budvicia* sp. | SB10 | *Bacillus* sp. | FB10 | Uncultured Syntrophaceae bacterium |
| MB11 | Uncultured bacterium | SB11 | Uncultured Acidobacteria bacterium | FB11 | Uncultured bacterium |
| MB12 | *Serratia* sp. | SB12 | *Clostridium* sp. | FB12 | Uncultured bacterium |
| MB13 | *Serratia* sp. | SB13 | *Acidobacterium* sp. | FB13 | Uncultured rumen bacterium |
| MB14 | *Serratia* sp. | SB14 | Uncultured bacterium | FB14 | Uncultured *Acidaminococcus* sp. |
| MB15 | *Enterobacter* sp. | SB15 | *Clostridium* sp. | FB15 | *Clostridium* sp. |
| MB16 | *Aeromonas* sp. | SB16 | Uncultured Solibacteraceae bacterium | FB16 | Uncultured *Clostridium* sp |

| MB17 | *Serratia* sp. | SB17 | Uncultured bacterium | FB17 | *Tissierella* sp. |
| --- | --- | --- | --- | --- | --- |
| MB18 | *Enterobacter* sp. | SB18 | *Nitrospira* sp. | FB18 | *Desulfovibrio* sp. |
| MB19 | *Aeromonas* sp. | SB19 | *Roseomonas* sp. |  |  |
| MB20 | *Klebsiella pneumoniae* | SB20 | *Castellaniella* sp. |  |  |
| MB21 | *Aeromonas* sp. | SB21 | Uncultured *Alcaligenes* sp. |  |  |
| MB22 | *Pantoea* sp. | SB22 | Uncultured Acidobacteria bacterium |  |  |
| MB23 | *Pantoea* sp. |  |  |  |  |
| MB24 | *Pantoea agglomerans* |  |  |  |  |

M: Swine meat; S: Farm soil; F: Swine feces; B: Bands


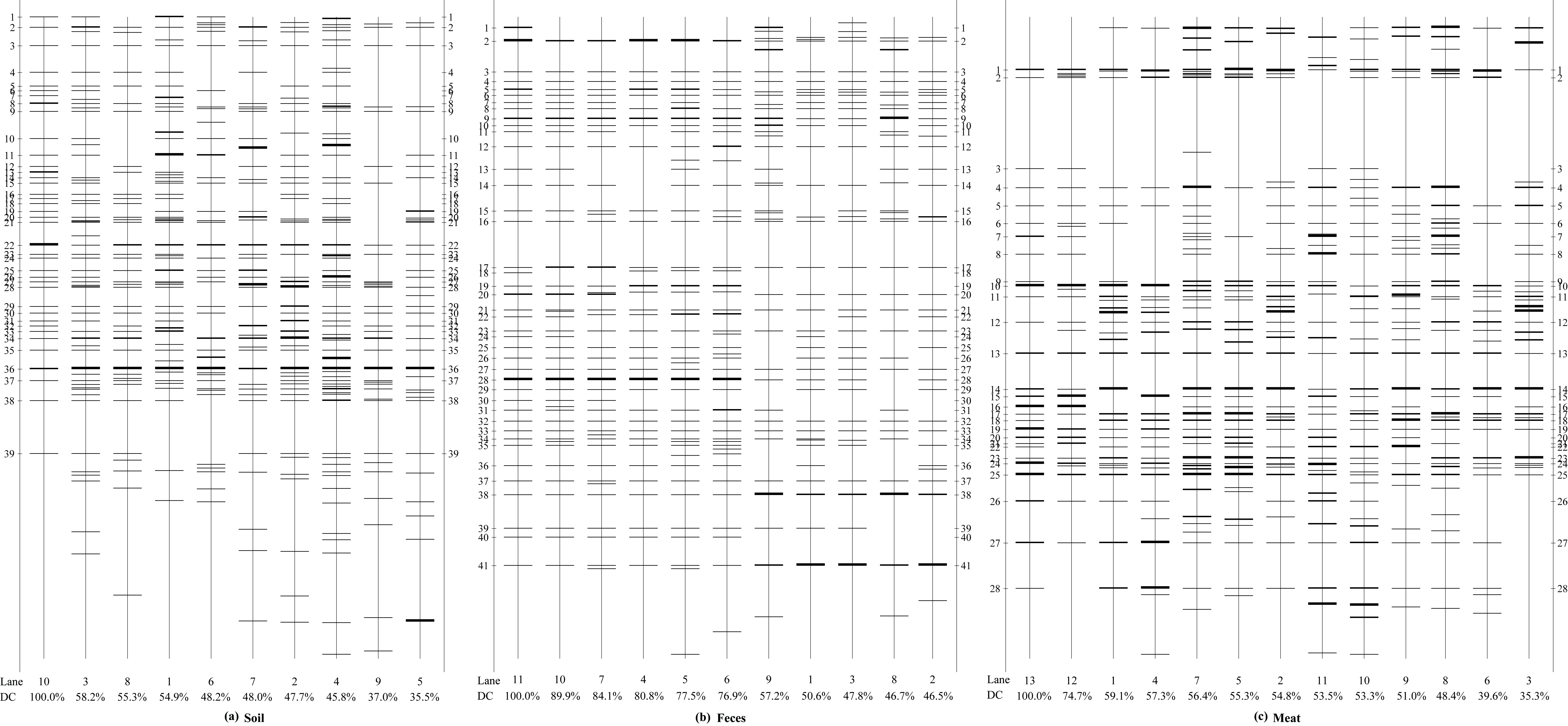


Fig. S1 Comparison of the dominant bacterial communities in environmental and meat samples. (a) Farm soil; (b) Swine feces; (c) Swine meat. DC: Dice coefficient. The analysis results of lane comparison from the DGGE patterns using Quantity One 4.6.2 software.

**Reference**

Jiang, X., and Shi, L. (2013). Distribution of tetracycline and trimethoprim/sulfamethoxazole resistance genes in aerobic bacteria isolated from cooked meat products in Guangzhou, China. *Food control* 30(1)**,** 30-34.

Lin, C.-H., Hou, R.F., Shyu, C.-L., Shia, W.-Y., Lin, C.-F., and Tu, W.-C. (2012). In vitro activity of mastoparan-AF alone and in combination with clinically used antibiotics against multiple-antibiotic-resistant Escherichia coli isolates from animals. *Peptides* 36(1)**,** 114-120.

Lv, L.-X., Hu, X.-J., Qian, G.-R., Zhang, H., Lu, H.-F., Zheng, B.-W., et al. (2014). Administration of Lactobacillus salivarius LI01 or Pediococcus pentosaceus LI05 improves acute liver injury induced by D-galactosamine in rats. *Appl. Microbiol. Biotechnol.* 98(12)**,** 5619-5632.

Mazurek, J., Bok, E., Pusz, P., Stosik, M., and Baldy-Chudzik, K. (2014). Phenotypic and genotypic characteristics of antibiotic resistance of commensal Escherichia coli isolates from healthy pigs. *B VET I PULAWY* 58(2)**,** 211-218.

Ng, L.-K., Martin, I., Alfa, M., and Mulvey, M. (2001). Multiplex PCR for the detection of tetracycline resistant genes. *MOL CELL PROBE* 15(4)**,** 209-215.

Van, T.T.H., Chin, J., Chapman, T., Tran, L.T., and Coloe, P.J. (2008). Safety of raw meat and shellfish in Vietnam: an analysis of Escherichia coli isolations for antibiotic resistance and virulence genes. *Int J Food Microbiol* 124(3)**,** 217-223.

Yamamoto, S., Iwabuchi, E., Hasegawa, M., Esaki, H., Muramatsu, M., Hirayama, N., et al. (2013). Prevalence and molecular epidemiological characterization of antimicrobial-resistant Escherichia coli isolates from Japanese black beef cattle. *J. Food. Prot.* 76(3)**,** 394-404.
